# Serum Procalcitonin Level as a Predictor of Bacterial Infection in Patients with COPD Exacerbation

**Published:** 2019-02

**Authors:** Atefeh Abedini, Arda Kiani, Habib Emami, Mohammad Hassan Touhidi

**Affiliations:** 1 Chronic Respiratory Diseases Research Center, National Research Institute of Tuberculosis and Lung Disease (NRITLD), Shahid Beheshti University of Medical Sciences, Tehran, Iran,; 2 Tracheal Diseases Research Center, NRITLD, Shahid Beheshti University of Medical Sciences, Tehran, Iran,; 3 Tobacco Prevention and Control Research Center, NRITLD, Shahid Beheshti University of Medical Sciences, Tehran, Iran.

**Keywords:** Procalcitonin, COPD, Exacerbation, Bacterial infection

## Abstract

**Background:**

Chronic Obstructive Pulmonary Disease (COPD) is a major cause of mortality and morbidity throughout the world. Although the cause of COPD exacerbations can be bacterial or viral, use of antibiotics in exacerbations remains controversial. Procalcitonin serum level dramatically increases in bacterial infections, but not in viral or noninfectious febrile diseases. The aim of this study is to investigate whether the measurement of procalcitonin can be used to differentiate bacterial from non-bacterial causes of COPD exacerbations.

**Materials and Methods:**

Sixty-eight COPD patients admitted to the emergency department of Masih Daneshvari Hospital due to COPD exacerbation were studied. At admission and before prescribing antibiotics, we obtained sputum and blood samples for sputum gram staining and culture and measured serum C-reactive protein and procalcitonin. All results were analyzed by SPSS software version 22.

**Results:**

A total of 68 patients including 51 males and 17 females were studied. From 38.2% of patients a respiratory pathogen was isolated from their sputum and 23.5% of patients had elevated serum procalcitonin values. Using Fisher exact test, we found strong correlation between elevated procalcitonin levels above 0.5 ng/ml and sputum culture results (P < 0.01). We also found strong correlation between elevated procalcitonin levels above 0.5 ng/ml with abnormal C-reactive protein levels in a group of patients with positive sputum culture, using Fisher exact test (P <0.01)

**Conclusion:**

As sputum culture and microbiologic studies are time consuming and sometimes expensive, it seems that procalcitonin could be a reliable marker of bacterial infection in COPD exacerbation, although we recommend a larger study with larger sample to consolidate the finding of this study.

## INTRODUCTION

Chronic Obstructive Pulmonary Disease (COPD), as it is currently defined, is a spectrum of lung abnormalities characterized physiologically by persistent airflow obstruction. COPD currently is the fourth leading cause of death in United States and is a major cause of mortality and morbidity throughout the world (1). It has been suggested that COPD will be the third cause of death in 2020 (2). More than 3 million people died of COPD in 2012 which accounts for 6% of all deaths globally (3). During the course of the disease there are periods of worsening symptoms called exacerbations. Exacerbations of COPD have major impact on disease control since they cause worsening of patient health, admission and readmission and disease progression (4). Exacerbations of COPD are complex events which are usually in correlation with increased airway inflammation, increased mucus and worsening of airflow obstruction beside increasing cough and wheezing. Since COPD accompanies other comorbidities such as coronary heart disease, congestive heart failure, pneumonia and pulmonary embolism, it is imperative to differentiate these diseases from acute exacerbations (5). The Global Initiative for Obstructive Lung Disease states, “An exacerbation of COPD is an acute event characterized by a worsening of the patient’s respiratory symptoms that is beyond normal day-to-day variations and leads to a change in medication” (5, 6).

COPD exacerbations are mostly due to respiratory viral infections, although bacterial infections and environmental factors such as air pollution and ambient temperature change can eventuate in worsening of symptoms (6). Rhinovirus is the most common isolated viral pathogen and can be detected up to a week after an exacerbation onset (6,7). Exacerbations can be associated with increased sputum production and, if purulent, some studies showed increased bacteria in sputum (7, 8).

Although the cause of COPD exacerbations can be bacterial or viral (9), use of antibiotics in exacerbations remains controversial (10, 11). Many studies tried to differentiate bacterial exacerbations from other causes of exacerbations in order to prevent widespread use of antibiotics. Traditionally positive bacteriologic sputum smear is considered a sign of bacterial exacerbations of COPD patients. But problems such as contamination of sputum with upper airway pathogens and inability to differentiate bacterial colorizations from infections drives researchers to pursue for alternate methods in order to confirm bacterial causes of exacerbations (12). Several biomarkers have been studied to evaluate infectious causes of upper respiratory infections which have more acceptable profile comparing to sputum culture. Currently C- reactive protein (CRP) and ESR are most common biomarkers to evaluate and monitor response to treatment of inflammatory and infections. Considering the fact that C-reactive protein can be elevated in both viral and bacterial infections, it lacks specificity to differentiate bacterial form viral infections (13, 14).

Procalcitonin, precursor of hormone calcitonin, is produced by C cells in thyroid and also by neuroendocrine cells in lung and intestine and its normal serum levels in healthy individuals is less than 0.1 ng/ml (15). Procalcitonin serum level dramatically increases in bacterial infections but not in viral or noninfectious febrile diseases (16, 17). Several studies showed superiority of procalcitonin as a diagnostic marker of bacterial infection relative to other biomarkers such as ESR and C-reactive protein (16,18, 19). Although procalcitonin levels of less than 0.5 ng/ml are contradicted with bacterial infections by most authors, evidence of infection has been seen with procalcitonin levels of less than 0.5 ng/ml (20).

The aim of this study was to investigate whether the measurement of procalcitonin can be used to differentiate bacterial from non-bacterial causes of COPD exacerbations, thus helping in treatment plan.

## MATERIALS AND METHODS

Sixty-eight COPD patients (51 men and 17 women) admitted to the Emergency Department of Masih Daneshvari Hospital due to COPD exacerbation were studied. Diagnosis of COPD was based on medical history and spirometric criteria according to Global Initiative for Chronic Obstructive Lung Disease (GOLD) guidelines. COPD exacerbation is defined as increased dyspnea, increased sputum volume or increased sputum purulence. Exclusion criteria included patients with immediate need to intubate, were unable to give a sputum sample or were finally diagnosed to have pulmonary emboli, pneumothorax, pneumonia and so on.

On every patient, we performed a complete medical history and physical examination including chest examination and obtained chest radiography. Demographic data inclusive of age, gender and smoking history were recorded. At admission and before prescribing antibiotics we obtained sputum and blood samples for sputum culture and measurement of serum C-reactive protein and procalcitonin. Sputum specimen with less than 10 epithelial cells and more than 25 leukocytes in low power field were considered acceptable. All blood samples were centrifuged and analyzed by immunofluorescence assay (Ichroma PCT kit, South Korea). Serum procalcitonin levels below 0.5 ng/ml were considered negative, were as levels equal or more than 0.5 ng/ml were considered positive (21). Ultimately all results were analyzed by SPSS software version 22.

## RESULTS

**Figure 1.**
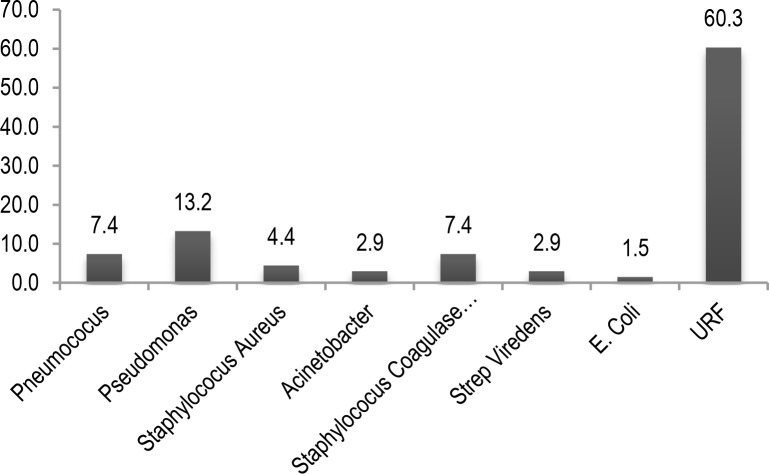
Sputum culture pathogens (percent) (URF-Usual Respiratory Flora).

A total of 68 patients including 51 males and 17 females were studied. The mean and standard deviation of the patients’ age were 65.25 and 10.54 years, respectively. Demographic data of the patients are shown in table 1. Sputum culture results, procalcitonin and C-reactive protein values are shown in table 2. In 38.2% of patients a respiratory pathogen was isolated from their sputum. *Pseudomonas aeruginosa* was the most common isolated pathogen with a prevalence of 13.2% and *Escherichia coli* (*E. coli*) was isolated from only one patient (Figure 1).

**Table 1.** Demographic data

Number of patients	68
Age(year):Mean ±SD	65.25±10.54
Male: N (%)	51 (75%)
Female: N (%)	17 (25%)
Smoking History(percent)	72.10
Antibiotic use in previous 3 months: N (%)	41(60.2%)
Prior admission to hospital due to exacerbation in past year: N (%)	32(47%)
Severity of COPD by GOLD criteria	
I (FEV1> or equal to 80% of predicted): N (%)	9(13.2%)
II (FEV1> or equal to 50% &<80%): N (%)	16(23.5%)
III (FEV1> or equal to 30% &<50%): N (%)	25(36.8%)
IV(FEV1< 30% of predicted): N (%)	18(26.5%)

**Table 2.** Serum procalcitonin and serum C-reactive protein levels

	Min.	Max.	Mean	Standard deviation
Procalcitonin (ng/ml)	0.10	23.00	1.29	3.53
CRP (mg/L)	0.70	68.00	10.14	14.08

**Figure 2.**
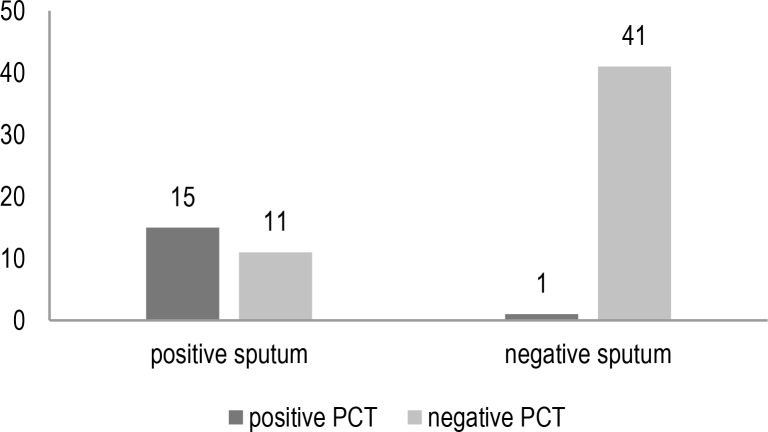
Comparing serum procalcitonin results in patients with positive or negative sputum cultures (Numbers)

**Figure 3.**
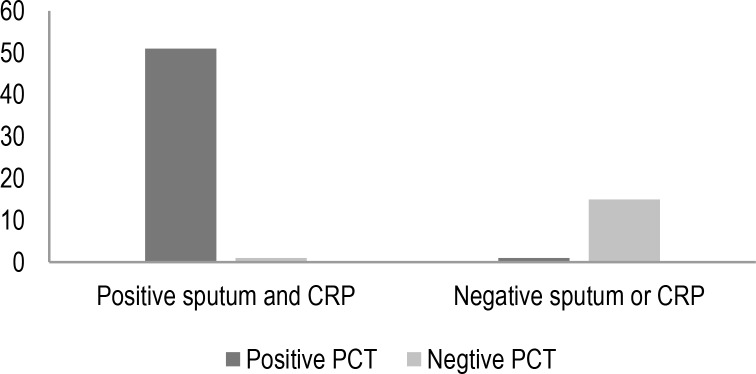
Comparing serum procalcitonin results in patients with or without concurrent positive sputum cultures and elevated CRP results (Numbers)

Figure 2 and 3 showed the comparison of serum procalcitonin results with positive or negative sputum cultures as well as elevated CRP results.

## DISCUSSION

In this study, we showed that the level of procalcitonin in patients with positive sputum culture for bacterial pathogens and concurrent elevation of C-reactive protein serum levels was significantly higher than that in patients with positive sputum culture alone or in patients with isolated elevation of serum C-reactive protein levels (P <0.01). Our findings were in line with those of some other studies in that patients with COPD exacerbation and positive sputum culture for bacterial pathogens had significantly higher serum procalcitonin levels (22,23). In another study conducted by Peng et al. on patients with COPD exacerbations caused by viral and bacterial infections, it was shown that inflammatory markers including serum C-reactive protein levels were considerably higher compared with patients with noninfectious causes of exacerbations (14). They also demonstrated that patients with bacterial exacerbations had a serum C-reactive protein level above 19.6 mg/L.

In contrast, Falsey et al. in a study performed on subjects with acute exacerbation of COPD concluded that serum procalcitonin does not distinguish bacterial from viral and noninfectious causes of acute exacerbation of COPD (24). In another study performed by Tanriverdi et al., it has been shown that although serum procalcitonin was better than C-reactive protein for predicting bacterial infection in hospitalized patients with acute exacerbation of COPD, serum procalcitonin was not so reliable in predicting bacterial infection in acute exacerbation of COPD due to sensitivity and specificity of less that 80% (25).

As a study by Rey et al. showed that procalcitonin threshold level of 0.5 ng/ml has a positive predictive value of 100% and negative predictive value of 87% for predicting bacterial infections (26), we believe that these differences can be explained by procalcitonin cut-off values in these studies, as those two aforementioned studies (24, 25), which considered procalcitonin values of >0.25 and 0.40 ng/ml as positive results, respectively. These findings are supported by a study performed by Christ-Crain et al. in which they have suggested that procalcitonin level of equal or more than 0.5 ng/ml is strongly associated with bacterial infection (21).

Our study has a limitation, since we could not properly differentiate patients with bacterial colonization from bacterial infections. As Sethi et al. studied 26 COPD patients and compared them with 20 smoker and 15 healthy people in 2006 in whom bronchoscopy and Bronchoalveolar Lavage (BAL) was performed. They demonstrated that patients with COPD may have tracheobronchial colonization with bacterial pathogen without any signs of exacerbations (12).

Because diagnosing bacterial infections are of great importance, procalcitonin seems an attractive test to diagnose bacterial infections since its serum levels are elevated as early as 3 to 4 hours after infection, which is much faster than other inflammatory markers such as ESR and C-reactive protein (27). For decades, guidelines recommend use of prophylactic antibiotic therapy in exacerbation of COPD based on Anthonisen criteria reported by patients and includes increased dyspnea, increased sputum volume or increased sputum purulence (28). Although prescribing antibiotics based on Anthonisen criteria is simple and practical, many authors believe that using these criteria results in antibiotic overuse without thorough microbiologic studies (12,29).

## CONCLUSION

Since sputum culture and microbiologic studies are time consuming and sometimes expensive, it seems that procalcitonin could be a reliable marker of bacterial infection in COPD exacerbation, thereby reducing antibiotic-related adverse reactions and decreasing antibiotic-resistant bacteria. Because of aforementioned limitation, we recommend a larger study with larger sample to consolidate the finding of this study.
